# Bi-directional association between female pattern hair loss and polycystic ovary syndrome: A systematic review and meta-analysis

**DOI:** 10.1016/j.jdin.2025.08.004

**Published:** 2025-08-25

**Authors:** Daniel G. Rayner, Eric McMullen, Kshitija Mundle, Michelle Pham, Cathryn Sibbald, Jeffrey Donovan

**Affiliations:** a Schulich School of Medicine and Dentistry, Western University, London, Ontario, Canada; b Division of Dermatology, Department of Medicine, Temerty Faculty of Medicine, University of Toronto, Toronto, Ontario, Canada; c Michael G. DeGroote School of Medicine, McMaster University, Hamilton, Ontario, Canada; d Cumming School of Medicine, University of Calgary, Calgary, Alberta, Canada; e Division of Dermatology, Department of Paediatrics, The Hospital for Sick Children, University of Toronto, Toronto, Ontario, Canada; f Department of Dermatology & Skin Science, University of British Columbia, Vancouver, British Columbia, Canada; g Donovan Hair Clinic, Whistler, British Columbia, Canada

**Keywords:** androgenetic alopecia, female pattern hair loss, infertility, meta-analysis, polycystic ovary syndrome, systematic review

*To the Editor:* Female pattern hair loss (FPHL) is a common form of hair loss affecting up to 38% of women.^1^ FPHL has previously been associated with medical conditions affecting fertility, including polycystic ovary syndrome (PCOS).^2^ However, the strength and nature of the association between FPHL and PCOS remain unclear. We conducted a systematic review and meta-analysis to evaluate the relationship between FPHL, PCOS and factors potentially associated with altered fertility.

We searched MEDLINE, Embase, and CINAHL from inception to December 4, 2024, to identify peer-reviewed observational studies evaluating the impact of FPHL on PCOS or fertility outcomes. Studies assessing the presence of FPHL in patients with PCOS were also included. A PROSPERO protocol was registered (CRD42025637699) and PRISMA reporting guidelines were followed. We used random effects meta-analyses to pool prevalences and odds ratios (ORs) and the GRADE approach to evaluate the certainty of the evidence. We used the STATA *metan* function for all analyses.

From 9243 citations, 24 observational studies met inclusion criteria. Of these, 6 studies reporting on 1583 patients (median age: 32.9 years) evaluated fertility measures or PCOS in patients with FPHL. The pooled prevalence of PCOS in patients with FPHL was 32.3% (13.6%-59.1%; Fig 1; very low certainty, due to serious risk of bias and very serious inconsistency). The pooled prevalence of irregular menstruation in patients with FPHL was 20.7% (13.4%-30.6%, 3 studies, I^2^ = 90.7%; very low certainty, due to serious risk of bias and very serious inconsistency). One study found that patients with FPHL were more likely to have polycystic ovaries compared to controls (OR: 5.48, 95%CI: 2.78-10.81).^3^

**Fig 1:**
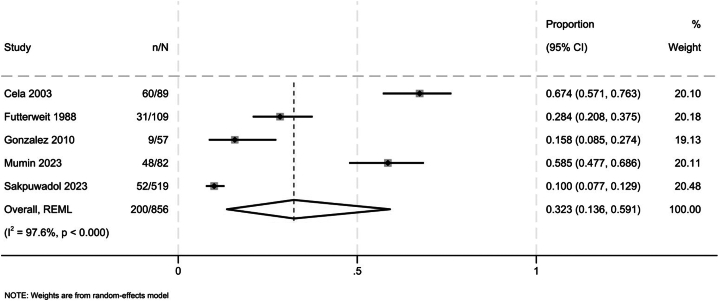
Forest plot presenting the prevalence of polycystic ovary syndrome in patients with female pattern hair loss.

Eighteen studies reporting on 3464 patients (median age: 25.5 years) assessed the prevalence of FPHL in patients with PCOS. The pooled prevalence of FPHL in patients with PCOS was 22.7% (16.9%-29.7%; Fig 2; low certainty, due to very serious inconsistency). Compared to controls, patients with PCOS had a higher risk of FPHL (OR: 4.74, 95%CI: 0.57-39.52, 2 studies, I^2^ = 55.2%; very low certainty, due to very serious risk of bias and serious inconsistency).

**Fig 2:**
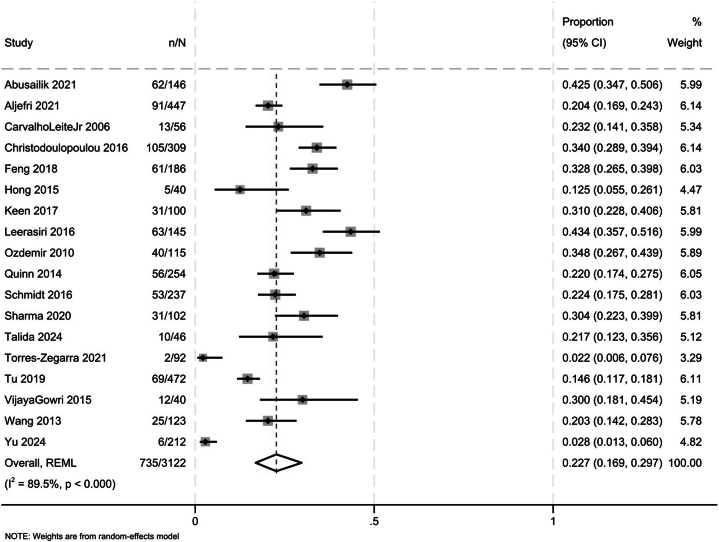
Forest plot presenting the prevalence of female pattern hair loss in patients with polycystic ovary syndrome.

Overlapping mechanisms of hyperandrogenism are known to be involved in the pathogenesis of PCOS and FPHL, and therapies frequently overlap when treating both conditions. PCOS is also associated metabolic syndrome,^4^ which may provide additional mechanisms contributing to FPHL.

Limitations of this review include the potential underdiagnosis of PCOS and FPHL in the included studies and their cross-sectional designs; future longitudinal studies are warranted to confirm the relationship between FPHL and PCOS. Additionally, we did not include studies evaluating patients with congenital adrenal hyperplasia without PCOS, who may also be at an elevated risk of FPHL.^5^

This is the first meta-analysis to summarize the bi-directional association between PCOS and FPHL. We found high rates of PCOS and menstrual irregularity in patients with FPHL, and high rates of FPHL in patients with PCOS. Our findings emphasize the importance of carefully evaluating menstrual regularity, signs and symptoms of hyperandrogenism, and fertility concerns in patients presenting with a diagnosis of FPHL.

## Conflicts of interest

Dr Cathryn Sibbald has received honoraria from Abbvie, Leo, Pfizer, Miravo, Novartis, UCB, Sanofi/Regeneron unrelated to this work. Dr Jeffrey Donovan has received honoraria from Pfizer and Vichy, has participated on advisory boards at Pfizer for payment, participates on the Board of Directors for the Scarring Alopecia Foundation, and has received royalties from UpToDate and is the active Director of the Evidence Based Hair Fellowship (EBHF) Training Program. No other authors have conflicts of interest relevant to this manuscript to disclose.
